# Diabetic Nephropathy without Diabetes

**DOI:** 10.3390/jcm4071403

**Published:** 2015-07-09

**Authors:** Katia López-Revuelta, Angel A. Méndez Abreu, Carmen Gerrero-Márquez, Ramona-Ionela Stanescu, Maria Isabel Martínez Marín, Elia Pérez Fernández

**Affiliations:** 1 Unidad de Nefrología, Hospital Universitario Fundación Alcorcón, C/ Budapest, 1, 28922 Madrid, Spain; E-Mails: aamendez@fhalcorcon.es (A.M.A.); Mimartinezm@fhalcorcon.es (M.I.M.M.); 2 Unidad de Anatomía Patológica, Hospital Universitario Fundación Alcorcón, C/ Budapest, 1, 28922 Madrid, Spain; E-Mails: CGuerrero@fhalcorcon.es (M.C.G.M.); Ristanescu@fhalcorcon.es (R.I.S.); 3 Unidad de Investigación, Hospital Universitario Fundación Alcorcón, C/ Budapest, 1, 28922 Madrid, Spain; E-Mail: eperezf@fhalcorcon.es

**Keywords:** diabetic nephropathy, idiopathic nodular glomerulosclerosis, hypertension, insulin resistance

## Abstract

Diabetic nephropathy without diabetes (DNND), previously known as idiopathic nodular glomerulosclerosis, is an uncommon entity and thus rarely suspected; diagnosis is histological once diabetes is discarded. In this study we describe two new cases of DNND and review the literature. We analyzed all the individualized data of previous publications except one series of attached data. DNND appears to be favored by recognized cardiovascular risk factors. However, in contrast with diabetes, apparently no factor alone has been demonstrated to be sufficient to develop DNND. Other factors not considered as genetic and environmental factors could play a role or interact. The most plausible hypothesis for the occurrence of DNND would be a special form of atherosclerotic or metabolic glomerulopathy than can occur with or without diabetes. The clinical spectrum of cardiovascular risk factors and histological findings support this theory, with hypertension as one of the characteristic clinical features.

## 1. Introduction

Nodular glomerulosclerosis was originally described by Kimmelstiel-Wilson [1] in 1936 as a lesion pathognomonic of diabetic nephropathy (DN). It was after many years that nodular glomerulosclerosis was described among people without diabetes [2]. Immunofluorescence, electron microscopy studies, and clinicopathological correlation are essential to differentiate other conditions that mimic nodular glomerulosclerosis, as for instance chronic membranoproliferative glomerulonephritis, dysproteinemias and organized glomerular deposition disease, and chronic hypoxic or ischemic conditions.

In 1999, Helzenberg [3] revised already published cases and established two different categories of patients with nodular glomerulosclerosis without diabetes. The first category concerns patients with impaired glucose metabolism (IGM) and/or retinopathy that were considered to have diabetic nephropathy (DN). The second one was named “Idiopathic Nodular Glomerulosclerosis (ING)” and it involved patients that did not show IGM but that had a histological pattern indistinguishable from DN.

Since then, reports of ING cases have appeared in the literature [4,5,6], many of them with some type of IGM, either impaired fasting glucose (IFG) or impaired glucose tolerance (IGT), with the oral glucose tolerance test (OGTT) due to the fact that diagnostic DM criteria are evolving to lower levels of fasting plasma glucose and HbA1C to make a diagnosis. In fact, some of these cases of ING would be diagnosed today as DM. Moreover, DN can be a form of diabetes onset and may have different types of presentation, as we and other authors have pointed out [7]. Therefore, differentiating between DN and DNND, is sometimes a difficult task.

Three main studies in the literature [8,9,10] and a previous review [11] demonstrate evidence in many cases where no IGM alteration has been detected, neither at biopsy nor in the follow up period of the patients. ING cases are mostly found in patients with hypertension, smokers and with overweight/obesity.

Current classification of DN establishes four types of DN [12] with glomerular changes: Class I, glomerular basement membrane thickening; Class II, mesangial expansion, mild (IIa) or severe (IIb); Class III, nodular sclerosis (Kimmelstiel-Wilson lesions); and, class IV, advanced diabetic glomerulosclerosis. This classification was understood as a way of describing the evolution of the disease from the lowest to the most advanced stage. However, it has not been validated from a clinical point of view and its etiopathogenic mechanisms remain unclear.

There is another aspect that has emerged during these years and that is the existence of diffuse glomerulosclerosis cases in patients without diabetes, which resembles class II DN [13].

For all these reasons, we will from here on refer to DN non Diabetes (DNND) as the group of nephropathies histologically indistinguishable from DN occurring in patients without diabetes, whether or not they present with IGM. We describe two cases and review all previously reported cases since it has been defined, in order to clarify some of the controversies around this entity.

## 2. Material and Methods

We describe two cases of nodular glomerulosclerosis indistinguishable from diabetic nephropathy with Kimmelstein-Wilson nodules in two patients without diabetes. From a total of 763 renal biopsies performed in our center between January 1998 and December 2014, 71 were diagnosed of diabetic nephropathy. From these, three belonged to non-diabetic patients. We had to discard one case in which we had no immunofluorescence study and the electron microscopy features suggested a thrombotic microangiopathy.

We performed a systematic review of ING published cases and series in the literature. We followed the recommendations of Cochrane guidelines [14] for Systematic Reviews of Interventions in those features where they could be applied. The aim of our study has been to redefine the entity known as ING and its associated risk factors. Our secondary aim was to establish a possible etiopathogenic hypothesis common to diabetic nephropathy. Eligibility criteria were idiopathic nodular glomerulosclerosis cases that included the necessary information to rule out diabetes according to current diagnostic criteria and other differential diagnosis.

We made a search in PubMed and Google using as key words idiopathic nodular glomerulosclerosis, nodular glomerulosclerosis without diabetes and diabetic nephropathy without diabetes. We also revised the bibliography from each of the articles encountered. Thirty-nine records published in Spanish or English were identified. In order to have homogeneity as to the diagnostic criteria and recorded data, records published before 1999 were excluded (*n* = 14). Twenty-two records were included [3,4,5,6,8,9,10,11,13,14,15,16,17,18,19,20,21,22,23,24,25,26,27], from 25 screened records. Three were excluded because they did not meet criteria for precise diagnosis. We built a database with the individualized information from all the series and cases, including our cases. Demographic, laboratory, clinical and histological available data were extracted. We considered as study variables the factors that are described in the literature as associated to the ING and other cardiovascular risk factors available in the series and case reports analyzed.

Some new variables were created. The estimated glomerular filtration rate (eGFR) was calculated by CKD-EPI creatinine equation [28] for all the serum creatinine values. Impaired fasting glucose (IFG) is defined by current criteria [29]: an elevated fasting plasma glucose concentration (≥100 and ≤125 mg/dL) and/or HbA1C between 5.7% and 6.4%. Diabetes mellitus is defined by an elevated fasting plasma glucose concentration above 125 mg/dL and/or HbA1C ≥6.5% [29]. The variable “impaired glucose metabolism” (IGM) comprises impaired fasting glucose, diabetes mellitus or impaired oral glucose tolerance test (OGTT). Regarding the body mass index (BMI) we considered overweight between 25 and 30 kg/m^2^ and obesity over 30 kg/m^2^. Proteinuria was divided into negative (0–0.029 g/day), albuminuria (0.03–0.3 g/day), mild (0.3–2.9 g/day) and nephrotic (>3 g/day).

We estimated the patients with metabolic syndrome in which we included the cases that presented simultaneously with IGM, arterial hypertension and obesity. In this case, a cut off BMI ≥25 kg/m^2^ was considered obesity for Asian people as has-been recommended by other authors [30] based on the excess morbidity and mortality risk above this value. We also considered the cases referred by the authors as metabolic syndrome although the criteria were not well specified.

We defined bad prognosis as chronic renal disease grade 4 or 5 at the moment of the biopsy [27] or the need for hemodialysis during the first year post biopsy.

### Statistical Methods

First we developed a descriptive and univariate analysis with the individual patient data. Results are described with counts and percentages in qualitative variables and mean, standard deviation and quartiles in quantitative data. Mean and standard deviation to age are computed, weighting by the sample size [31].

To study factors associated with a poor prognosis, a univariate analysis was performed. Chi-square test or Fisher exact test and *t*-Student test was applied. Odds ratio (OR) and 95% CI were estimated by univariate logistic regression.

We used meta-analysis methods to estimate the pooled prevalence of risk factors [32]. Series with more than five cases [8,9,10,13] were included in this analysis. Pooled prevalence was estimated with fixed effect models and random effects models in case of high heterogeneity. Heterogeneity was evaluated with heterogeneity index I^2^.

In statistical analysis were used SPSS 17, MS Excel 2007 and meta-analysis tool MetaXL, developed by Epigear International Pty Ltd.

## 3. Case Report

### 3.1. Case 1

A 74 year-old male had a history of high blood pressure with bad control for 20 years, and dyslipidemia. He smoked 40 cigarettes a day until he was 60 years old, but did not fulfill criteria for chronic obstructive pulmonary disease. He had an aortic bifemoral bypass in 1995 and ischemic cardiomyopathy revascularized in 1998. Four years previously, nephrology analysis diagnosed probable nephroangiosclerosis (serum creatinine of 1.7 mg/dL, albuminuria/creatinine ratio 50 mg/g, normal sized kidneys with increased cortical echogenicity and normal renal Doppler). He had no family history of diabetes and was in treatment with Atenolol, Diltiazem and Clortalidona. He was admitted to the hospital in 2004 with a non-oliguric acute progressive renal failure just a few days after suffering an anterolateral myocardial acute infarction that was treated with fibrinolysis with Tenecteplasa. On admission his blood pressure was 153/77 mmHg, his BMI was 31 kg/m^2^, he had an increased waist circumference and the rest of the physical exam was normal. The serum creatinine increased progressively from 3.2 to 5.9 mg/dL. Diuresis was preserved and he had proteinuria of 1.4 g/day with no microhematuria or leukocyturia. Other laboratory tests were: glucose 112 mg/dL, urea 257 mg/dL, serum albumin 2.8 g/L, uric acid 11.9 mg/dL, hemoglobin 12.7 g/dL, total cholesterol 172 mg/dL, LDL cholesterol 132 mg/dL and triglycerides 150 mg/dL. HBV, HCV and HIV serology was negative. A new renal Doppler ultrasound study and a DMSA Cortical Renogram were performed and did not find signs of impaired renal vasculature. Other possibilities that were considered responsible for AKI in this patient were cholesterol crystal atheroembolism or acute tubular necrosis.

**Figure 1:**
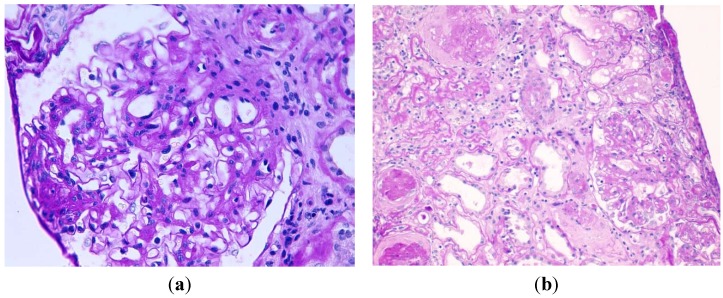
(**a**) Nodular mesangial expansions by acellular, PAS positive material, global thickening of glomerular basement membranes, and glomerular hypertrophy. There is an arteriole without hyalinosis (periodic acid-Schiff, original magnification ×400); (**b**) Another area with glomerular sclerosis, atrophic tubules, interstitial fibrosis, and arteriolosclerosis with hyalinosis (periodic acid-Schiff, original magnification ×200).

A renal percutaneous biopsy was performed. Three cores of renal cortex and medulla were obtained. They included 23 glomeruli, 16 of which showed global sclerosis (69.5%). The remaining seven glomeruli showed increased mesangial matrix and focal formation of characteristic nodules (Figure 1). The material in the nodules and mesangium was strongly PAS, Masson and Jones methenamine silver positive. Thioflavin stain was negative, excluding amyloid deposits. The capillary lumina were often narrowed and the glomerular basement membranes were focally thickened. Inside a glomerular capillary a cholesterol crystal was observed. There were no ischemic changes in the glomeruli nor data of thrombotic microangiopathy. No microaneurysms or mesangiolysis or exudative/insudative lesions were observed. Hyalinosis of arterioles, including those at the vascular pole, was seen. In interlobular arteries, mild intimal/mural sclerosis was also found. Moderately focal tubular atrophy with basement membranes moderately thickened and interstitial fibrosis existed. Immunofluorescence study revealed mild focal granular deposits of IgG and IgA in the glomerular basement membranes, and very scant deposits of IgM, K y L in the mesangium. CD34 inmunohistochemistry showed patent glomerular capillaries surrounding mesangial nodules. Electron microscopy showed no representative material. Though nephroangiosclerosis was clinically suspected the existence of nodular lesions and predominance of arteriolar hialinosis over sclerosis supported the diagnosis of nodular glomerulosclerosis. The absence of capsular adhesions discarded focal segmental glomerulosclerosis. Other diagnoses were ruled out in this patient: monoclonal immunoglobulin deposition disease (there were no deposits of Kappa or lambda chains), membranoproliferative glomerulonephritis (absence of C3 deposits), amyloidosis (abscense of amyloid), fibrillary and immunotactoid glomerulonephritis (absence of deposits of IgG or C3). Intraglomerular atheroembolism was confirmed.

One month after the renal biopsy the patient was readmitted with acute gastroenteritis and severe acute renal failure. His serum creatinine was 12 mg/dL and he required dialysis thereafter until his death in 2009. Monthly levels of glycemia had remained below 110 mg/dL up until two years after the renal biopsy when he presented some increased fasting glycemia levels up to 180 mg/dL and several HbA1c levels of 4.5%–5.7%. There was no fundoscopy performed. This was considered a case of advanced DNND with superimposed cholesterol atheroembolism in a patient with metabolic syndrome.

### 3.2. Case 2

A 53 year-old male had a history of multiple drug abuse since 15 years of age, initially with intravenous heroin and lately with cocaine and alcohol dependence. He was HIV positive stage B-3 since 25 years of age with minimum CD4 levels of 170/mL (10%). He was treated in another hospital with various antiretroviral drugs and since March 2011 he had a good immunoviral response to Raltegravir and Lopinavir/rtv (CD4 of 655/mL = 18% and undetectable viral load). He smoked 40 cigarettes a day since 13 years of age with no COPD criteria. His blood pressure control was quite good under treatment with spironolactone 100 mg per day, he suffered from hypertriglyceridemia, asymptomatic hyperuricemia and chronic hepatopathy with HCV, genotype 1A. His serologic test revealed past HBV and HAV infections. Mantoux test was repeatedly negative. He had chronic kidney disease for the last four years with basal creatinine of 1.6–1.8 mg/dL, albuminuria/creatinine ratio of 30 mg/g and proteinuria/creatinine ratio of 0.3–0.5 g/g without microhematuria. Several cryoglobulin determinations had been negative, antinuclear antibodies were positive at titers of 1:80, immunoglobulins and complement C3 and C4 were in normal range. The serum and urine proteinogram and immunofixation did not reveal any monoclonal bands.

The patient was admitted with abdominal distention treated outside the hospital with diuretics and worsening of his CKD with a maximum creatinine level of 4.5 mg/dL, eGFR (CKD-EPI) of 13.9 mL/min/m^2^, proteinuria/creatinine ratio of 0.65 g/g, microhematuria of 50–100 RBC/HPF, serum glucose of 98 mg/dL, albumin of 3 g/dL, uric acid of 12.5 mg/dL, LDL cholesterol of 108 mg/dL, triglycerides of 419 mg/dL and HbA1C of 5.2%. The immunological study was still normal. On admission he had a blood pressure of 125/69 mmHg, his BMI was of 24.6 kg/m^2^ and he was suspected of non-tensional ascites with no peripheral edemas. An abdominal CT was performed which confirmed signs of chronic liver disease and a mild bilateral renal cortical atrophy and discarded ascites and other signs of portal hypertension. A multifactorial chronic kidney disease due to cocaine abuse vascular changes or highly active antiretroviral therapy (*HAART*) renal toxicity superimposed on an HIV or HCV associated glomerulonephritis was suspected.

**Figure 2:**
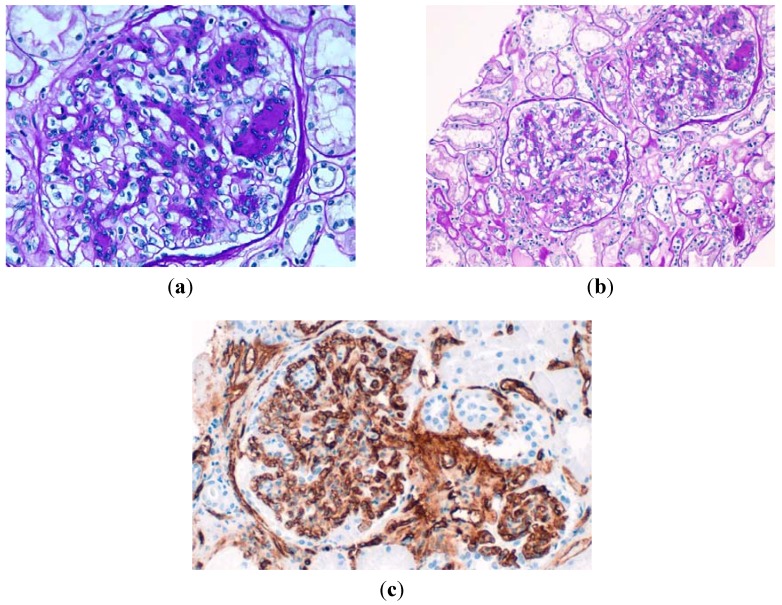
(**a**) Glomerulus displays less-prominent mesangial sclerosis with early nodularity by acellular, PAS positive material and glomerular hypertrophy. (Periodic acid-Schiff, original magnification ×400); (**b**) A lower-power view that shows two glomeruli with nodular sclerosis, an arteriole without hyalinosis and mild atrophic tubules and interstitial fibrosis (periodic acid–Schiff, original magnification ×200); (**c**) The mesangial nodules were surrounded by patent glomerular capillaries (inmunohistochemestry with CD34 original magnification ×400).

A percutaneous renal biopsy was performed. One core of renal cortex and medulla was obtained. It included 12 glomeruli, three of which showed global sclerosis (25%). The remaining eight glomeruli showed increased mesangial matrix and cellularity and focal formation of characteristic nodules (Figure 2). Thioflavin stain was negative. The capillary lumina were focally thickened and there were some capsular adhesions. No duplication of the glomerular basement membrane, nor crescent formations were observed. A mild hyalinosis of arterioles, including those at the vascular pole, was seen. In interlobular arteries, mild intimal/mural sclerosis was also found. Mild focal tubular atrophywith basement membranes moderately thickened and mild interstitial fibrosis existed. No hyperplasia of podocytes nor vascular collapse in the glomeruli was observed. There were no extra- or endocapillary proliferation, vascular permeation by inflammatory cells, nor capilar thrombosis. At tubulointerstitial level only a very isolated patch of tubular atrophy with mild chronic inflammatory infiltrate was observed which ruled out an important role of HAART toxicity. Immunofluorescence in a core with 10 glomeruli with sclerosis of six, has shown little unspecific, segmental and focal deposits, mainly of C3 in the mesangium, Bowman capsule, tubular basement membranes and some arterioles. CD34 inmunohistochemistry showed patent glomerular capillaries surrounding mesangial nodules. Electron microscopy showed an increase of the mesangial matrix, slightly diffuse thickening of the glomerular basement membranes and effacement of foot processes with absence of deposits. Although some of the glomerular changes could be found in relation to HIV-associated nephropathy (focal and segmental glomerulosclerosis), they do not correspond to the collapsing form, and they are not accompanied by other usual findings such as IgM and C3 deposits or tubular microcysts. Crioglobulinemic glomerulonephritis and membranoproliferative glomerulonephritis were also discarded by the absence of characteristic histological picture and immunofluorescence data. There was no data of cocaine abuse damage (hypertensive nephrosclerosis, malignant hypertensive nephropathy), nor tubulointersticial nephritis. The histological diagnosis was diffuse glomerulosclerosis with some images suggestive of nodular glomerulosclerosis (Kimmelstiel-Wilson) supported by immunofluorescence and electron microscopy studies.

Because of the renal biopsy findings, we re-evaluated his glucose metabolism. The patient had a family history of diabetes. Lab results from previous years showed fasting glucose levels between 100–110 mg/dL. However, several determinations of HbA1c were below 5.7% and the fundoscopy discarded the diagnosis of diabetes. In the light of this data a diagnosis of DNND was made. The patient met criteria for metabolic syndrome, we do not know if protease inhibitors could have played a role in its development. He gradually recovered renal function with creatinine levels of 2 mg/dL and since then he has been followed in a nephrology clinic. Two years after the biopsy he had no signs of diabetes, nor hypertensive retinopathy, his Hb1Ac remained at ≤5% and despite four years of continued smoking and several relapses in cocaine consumption, his levels of creatinine and proteinuria remained stable. He was treated with spironolactone since the beginning of monitoring and Allopurinol since the renal biopsy.

## 4. Results

Including the two patients reported in this work, there are 95 cases of diabetic nephropathy without diabetes (DNND) described in the literature between 1994 and 2015 (Table 1).

**Table 1:** Case reports of Diabetic Nephropathy non Diabetes (DNND).

Year	Author	Country	Number of Cases	Race	Sex = M	Mean Age	HTA	Smoker	Obese	Impaired Glucose Metabolism
1999	Herzenberg A.M.	Canada	2	White	2	64.5	2		2	1
2001	Grcevska L.	Macedonia	3		3	54.5	2			3
2002	Muller-Hocker J.	Germany	1		1	45	1	0	0	1
2002	Altiparmak M.R.	Turkey	1		0	31	1	0	0	1
2002	Markowitz G.S.	USA	23	White: 17 Black: 6	18	68.2	22	21	3	11
2005	Navaneethan S.D.	USA	1	White	0	66	1	0		0
2005	Chang C.	Taiwan	3	Asian	0	72	3	0	0	2
2006	Sánchez J.C.	Spain	2		2	47.5	1	2	0	1
2006	Kuppachi S.	USA	1	White	0	77	1	1		0
2006	Kusaba T.	Japan	3	Asian	3	69	3	3	0	1
2007	Nasr S.H.	USA	1	White	0	70	1	1		0
2007	Sanai T.	Japan	7	Asian	6	57	6	3	1	7
2007	Liang K.V.	USA	1	White	0	66	1	1	0	0
2008	Li W.	USA	15	White: 12 Black: 3	5	64.2	14	10	9	13
2009	Kasmani R.	USA	1		0	82	1	0		0
2009	Helai I.	Tunisia	1	White	1	49	0	0		
2011	Pedrosa A.F.	Brazil	1	White	1	65	1	1	0	0
2011	Kinoshita Ch.	Japan	1	Asian	1	72	1	1	0	1
2012	Uchida T.	Japan	1	Asian	0	53	0	0	0	0
2014	Batal I.	USA	3	White	2	52.3	3	3		0
2014	Filippone E.J.	USA	1	Black	0	27	0	0	1	1
2014	Wu Y.	Japan	20	Asian	16	50.5	19	17	8	6
2015	López-Revuelta K.	Spain	2	White	2	63.5	2	2	1	2

Individual patient data was available in all documents except in Markowitz’s series [8] where information has been published as aggregated data. Clinical findings at kidney biopsy are summarized in Table 2. The DNND published cases are more frequently males and Caucasian and Asian patients. The mean age at onset is 60.5 ± 13.7 years, older in Caucasians than in African-Americans and Asians but without statistically significant differences (data not shown in table). According to our classification, 56.7% of the cases showed some glucose metabolism alteration at the moment of the biopsy and in 42% of the cases these alterations were definitely ruled out. Only two patients could be classified as diabetics by current criteria. On the other hand, out of 44 patients who had a fundoscopy performed, five presented diabetic retinopathy, all with impaired OGTT and one of them diabetic.

**Table 2:** Clinical Findings at kidney biopsy.

Clinical finding	Estimate	Value	*n* Cases with Available Data
Mean Age at Biopsy	(years) (range)	60.5 ± 13.7 (16–84)	95
Gender	M/F	63/32	95
Ethnicity	% White	47.7	88
	% Black	11.4	
	% Asiatic	40.9	
Family history of diabetes	%	12.3	73
Smokers	%	73.3	90
History of Hypertension	%	87.4	95
Hypertension	%	90.5	95
Dislipemia	%	71.9	64
BMI	% Normal	30.6	62
	% Overweight	32.3	
	% Obese	37.1	
Impaired fasting glucose	%	45.2	90
OGTT *	% Abnormal	34.5	55
HB A1C	% Diabetes ≥6.5	2.8	71
	% Abnormal 5.7–6.4	19.7	
	% Normal <5.7	77.5	
Metabolic Syndrome ^&^	%	37.7	69
Serum Creatinine	mg/dL	3.1 ± 1.9	95
CKD-EPI eGFR (mL/min/m^2^)	% ERC 1–2	11.6	95
	% ERC 3	24.6	
	% ERC 4 % ERC 5	33.3 30.4	
Proteinuria g/day		3.9 ± 2.9	95
Degree of Proteinuria	% negative % 0.03–0.29 % 0.3–2.9	1.1 0 37.9	95
	% nefrotic ≥3	61.1	
Months of Proteinuria	Median (IQR)	24 (3.5–30)	13
Diabetic Retinopathy	(%)	11.4	44
Hypertensive Retinopathy	(%)	17.1	41

* OGTT: Oral Glucose tolerance test; ^&^ Metabolic Syndrome: cases referred to by the authors as well as those patients who exhibited simultaneously abnormal metabolism of glucose + hypertension + obesity.

Eighty-eight percent of the patients were known to have high blood pressure and this percentage rose to 90.5% at the moment of the biopsy. Seven of 41 cases exhibited features of hypertensive retinopathy on fundoscopy, all but one previously hypertensive. Thirty-three out of the 35 cases in which we had information about the antihypertensive therapy received rennin-angiotensin-aldosterone system inhibitors (RAASI) although we do not know the duration of the treatment.

Seventy three percent of the patients were or had been smokers and a similar percentage (72%) suffered from dyslipidemia. Sixty nine percent of the cases presented overweight-obesity and 37% were obese. The percentage of the subjects with metabolic syndrome was 37.7% (95% IC: 24.2%–48.3%).

The clinical presentation is that of a proteinuric renal failure with a mean creatinine of 3.1 ± 1.9 and proteinuria of 3.9 ± 2.9 g/d, at nephrotic levels in 61% of the cases. Sixty-four percent of the patients presented at diagnosis with CKD stage 4–5 and 52% of them required dialysis after a median (IQR) 1 (1–3.5) years. Other laboratory data not shown in the table are: microhematuria in 25 out of 30 cases, hyperuricemia/gout in 12 out of 33 cases (37%), peripheral vasculopathy in 14 out of 42 patients (33%), hypoalbuminemia in nine out of 44 patients (21%) and three out of 54 patients (5.6%) were positive for HCV.

As to the histological data, 91 cases were diagnosed with nodular glomerulosclerosis (class III) and four cases with class II (2 cases IIb and 2 cases IIa). In all of the cases arteriolar hyalinosis or sclerosis were reported (moderate to severe in 86.4% of the cases). In 58% of the cases there was moderate to severe tubulointerstitial fibrosis and tubular atrophy (Table 3).

**Table 3:** Histopathological findings.

Histopahological Finding	Degree	*n*	Frequency	95% CI	
Glomerular Class	Mesangial expansion *	95	5.3%	1.7%	11.9%
	Nodular Sclerosis		95.8%	86.9%	98.4%
Interstitial fibrosis and tubular atrophy	No	86	3.5%	0.7%	9.9%
	<25%		38.4%	28.6%	50.5%
	25%–50%		32.6%	21.0%	41.8%
	>50%		25.6%	15.8%	35.4%
Arteriolar Hyalinosis/sclerosis	Light	81	13.6%	6.8%	22.8%
	Mild-severe		86.4%	78.3%	94.5%

* Previously called “diffuse diabetic glomerulosclerosis”.

Table 4 shows results from univariate analysis. Both tubulointerstitial fibrosis and tubular atrophy (IFTA) and arteriolar hyalinosis or sclerosis (AH/S) are risk factors of poor renal prognosis. Odds ratio IFTA 2-3/0-1 is 16.5 (95% CI: 4.3 to 62.7, *p* < 0.001) and odds ratio AH/S 2-3/0-1 is 7.6 (95% CI: 1.7 to 34.6, *p* = 0.008). Also patients with history of hypertension have a worse prognosis than no previous renal hypertensive, OR = 5.1, (95% CI: 1.1 to 23, *p* = 0.033).

**Figure 3:**
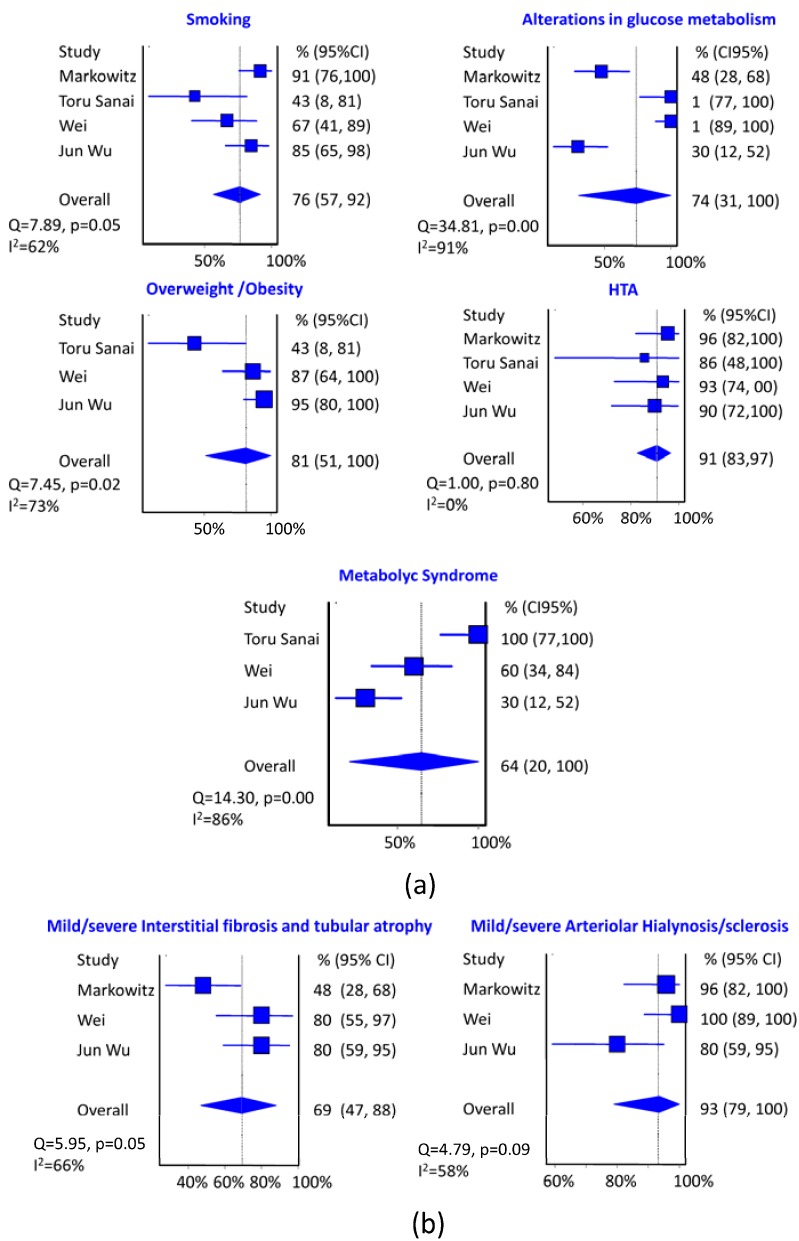
(**a**) Summary of estimates (95% CI) of the prevalence of DNND clinical risk factors. The size of the blue boxes is directly proportional to the weight assigned to each study; (**b**) Summary of estimates (95% CI) of the prevalence of DNND histological risk factors. The size of the blue boxes is directly proportional to the weight assigned to each study.

In the meta-analysis it is clear that the most important risk factor studied to suffer DNND is hypertension with a pooled prevalence of 91% (95% CI: 83% to 97%, I^2^ = 0%) (Figure 3). Secondly, overweight/obesity with a pooled prevalence of 82% (95% CI: 51% to 100%, I^2^ = 73%), although in this case there is considerable heterogeneity in the results of the different series. Smoking and alterations in glucose metabolism occupy the third place with a pooled prevalence about 75% although alterations in glucose metabolism have a very high heterogeneity in different series. The effect of metabolic syndrome reaches 64%, although with very high heterogeneity.

**Table 4:** Univariate analysis of possible prognostic factors.

Univariate Analysis		Bad Prognosis *n* (%)	*p*-Value
Race	Asian	23 (69.7%)	0.098
	White	19 (79.2%)	
	Black	1 (25%)	
Sex	Female	19 (73.1%)	0.373
	Male	25 (62.55)	
Smoker	No	14 (70%)	0.78
	Yes	29 (64.4%)	
Hypertension History	No	3 (33.3%)	0.051
	Yes	41 (71.9%)	
Overweight/Obesity	No	13 (68.4%)	0.609
	Yes	24 (61.5%)	
Dyslipemia	No	11 (68.8%)	0.742
	Yes	15 (60%)	
Metabolic Syndrome	No	26 (63.4%)	0.542
	Yes	17 (70.8%)	
IGM ^&^	No	16 (66.7%)	0.942
	Yes	25 (67.6%)	
Proteinuria (g/day)	No-minor	22 (73.3%)	0.294
	Nefrotic	22 (61.1%)	
Peripheral vasculopathy	No	5 (33%)	0.262
	Yes	3 (72%)	
Interstitial Fibrosis and Tubular atrophy	0	1 (33.3%)	<0.001
	1	5 (27.8%)	
	2	18 (81.8%)	
	3	15 (93.8%)	
Arteriolar hyalinosis/Sclerosis	1	3 (30%)	0.023
	2	26 (74.3%)	
	3	10 (83.3%)	
Age *	Bad prognosis	60.3 ± 15.8	0.162
	Good Prognosis	54.5 ± 15.9	
Proteinuria *	Bad prognosis	3.6 ± 2.8	0.584
	Good Prognosis	3.9 ± 2.1	
eGFR *	Bad prognosis	16.3 ± 6.5	<0.001
	Good Prognosis	58.7 ± 29.9	

^&^ Impaired Glucose Metabolism; * Mean ± standard deviation.

The most frequent histological data in DNND is arteriolar hyalinosis/sclerosis with a pooled prevalence of 93% (95% CI: 79% to 100%, I^2^ = 58%), compared to the tubulointerstitial fibrosis and tubular atrophy moderate/severe with a pooled prevalence of 69% (95% CI: 47% to 88%, I^2^ = 66%). In both cases considerable heterogeneity between studies is presented.

The GFR pooled average at the time of renal biopsy is 25 mL/min/m^2^, (95% CI: 19.4 to 30.7, I^2^ = 0%) evenly in all series. CKD stage at diagnosis is 4–5 with a pooled prevalence of 68% (95% CI: 54% to 82%, I^2^ = 0%) and nephrotic proteinuria is presented with a pooled prevalence of 61%(95% CI: 42% to 79%, I^2^ = 57%).

## 5. Definition and Terminology

We believe that the term idiopathic nodular glomerulosclerosis should be abandoned based on the cases of diffuse glomerulosclerosis [4,6,26] that have been described in patients with similar characteristics to those described with ING [3]. Although diffuse glomerulosclerosis cases have been much less referred, it is not unusual because the definition of ING itself selects the cases that have been published.

We also believe that DNND is a continuous and progressive process with different stages, in which, even in its advanced stage, it is indistinguishable from DN. Regarding this, the lesion described as isolated ultrastructural diffuse thickening of the glomerular capillary basement membrane [33] (corresponding to Class 1 DN), that the authors described as a pre-diabetes lesion, also appears in 30% of patients without any evidence of diabetes later on.

We do not consider adequate other terms previously proposed such as “smoking associated nodular glomerulosclerosis” [13] or “normoglycemic diabetic nephropathy” [27] because they do not reflect the spectrum of factors associated with the DNND that are acknowledged nowadays.

We found more appropriate the term DNND or diabetes like nephropathy to refer to the indistinguishable histologic entity from diabetic nephropathy in any of its types, which appear in subjects without diabetes.

However, the possibility of speculating with a wider term that included diabetic nephropathy of non-diabetic and diabetic patients based on common etiopathogenic mechanisms like “aterosclerotic microangiopathy or metabolic microangiopathy” is not appropriate until the etiopathogenic mechanisms for both diseases are better understood.

## 6. Histological Findings

Most published DNND cases show different degrees of increase in mesangial matrix with nodule formation accompanied by glomerular basement membrane (GBM) thicknening that cannot be distinguished from diabetic nodular glomerulosclerosis (Class III DN) by routine light microscopy, immunofluorescence and electron microscopy. There is absence of immune deposits in immunofluorescence and linear staining of the GBM and tubular basement membranes for immunoglobulin G, and albumin is generally present. As mentioned before, although there is selection bias by definition and the search bibliography process, 96% of referred cases are nodular glomerulosclerosis and only 4% correspond to diffuse mesangial sclerosis (class IIa and class IIb of DN current classification) [4,6,16]. As far as we know, we are the first authors speculating about the fact that isolated thickening of GBM considered as a pre-diabetes lesion could correspond to an earlier stage of DNND (class I DN).

Some additional features of DN such as capsular drop lesions, glomerular hyalinosis, areas of mesangiolysis, and microaneurysms may also be observed. Different degrees of glomerulomegaly have been described in some studies [8,10] but not in others [27] nor in our study.

Another characteristic feature present in 100% of the reported cases is arteriolar hyalinosis/sclerosis, with a pooled prevalence of 93% (95% CI: 79% to 100%, I^2^ = 58%) and a moderate heterogeneity between series (Figure 1b). Renal arteriolar hyalinization is a uniquely human finding characterized by replacement of lost arteriolar cells by blood-derived proteinaceous materials in a manner akin to thrombosis but without participation of important amounts of platelets. It is considered a renovasculopathy of hypertension associated with diabetes, age and probably smoking. Renal arteriolar hyalinization correlates very strongly with coronary heart disease and it has been postulated that share common pathogenic mechanism with atherosclerosis [34]. In a previous review of 42 ING cases published until 2006, Kusaba *et al.* [18] found 90% arteriolosclerosis. The authors speculated that although the mechanisms of both afferent and efferent arteriolosclerosis in ING remain unclear, prominent arteriolar hyalinosis, mainly by its hemodynamic consequences, may play a preceding role in the progression to nodular formation. They concluded that ING is one of the phenotypes of renal arteriosclerosis without DM.

Tubulointerstitial fibrosis and tubular atrophy of mild-severe degree have been observed in 58% of the cases. Their pooled prevalence is 69% (95% CI: 47% to 88%, I^2^ = 66%) with considerable heterogeneity between studies.

Until now the histological features associated with renal prognosis have been the interstitial fibrosis [8,10], the arteriosclerosis [8] and the arteriolar hyalinosis although the latter is not acknowledged in all the cases. In our analysis the tubulointerstitial fibrosis and tubular atrophy (IFTA) and the arteriolar hyalinosis/sclerosis (AH/S) are very important prognostic factors. The IFTA has an Odds Ratio (OR) (95% CI) of 16.5 (4.3–62.7) *p* < 0.001 and the AH/S an OR ( 95% CI) of 7.6 (1.7–34.6) *p* < 0.008.

An interesting finding first described by Markowitz *et al.* [8] is the immunohistochemical staining for the endothelial marker CD34 at the mesangial nodules which appeared to contain increased endothelial-lined vascular spaces, suggesting a potential mechanism of neovascularization. They found intranodular CD 34 positivity in all the 10 cases tested that has been corroborated by others and us [9,10,16] although not in every case [10].

Also a potential pathogenic role of AGE is supported by immunohistochemical staining for pentosidine in the mesangial nodules and zones of interstitial fibrosis with an identical pattern in DNND and DN [8,13]. The same authors also described immunohistochemical staining for VEGF in the distribution of podocytes with segmental loss in areas of prominent sclerosis in ING, identical to that seen in cases of nodular diabetic glomerulosclerosis while normal controls displayed diffuse podocyte positivity for VEGF and complete negativity for pentosidine.

From these data we can deduce that angiogenesis is one of the pathogenetic mechanisms involved in DNND. It is unclear whether this glomerular angiogenesis is a response to hemodynamic stimuli or altered expression of endothelial growth factors.

## 7. Clinical Features

Most of the cases described are mild to severe CKD stages with nephrotic range proteinuria, half the cases needing dialysis in a short period of time, median (QI): 1 (1–3.5) year. The GFR pooled average at the time of renal biopsy is 25 mL/min/m^2^, (95% CI: 19.4 to 30.7, I^2^ = 0%) evenly in all series. It seems that in the case of Caucasians, the prognosis has been worse than in Asians, with an OR of 1.7 (95% CI: 0.5 to 5.7), although not in a significant way.

Hypertension is almost a constant feature with a pooled prevalence of 91% (95% CI: 83% to 97%, I^2^ = 0%) and microhematuria is frequently referred.

A rapidly progressive onset similar to DN has also been observed by us and others.

We could imagine that if early diagnosis would have been possible, its natural history, probably would have not differ from that of DN, with the typical early clinical presentation of albuminuria that increases with time to less selective proteinuria, with the eventual development of nephrotic-range proteinuria in many patients, usually associated with a decrease in glomerular filtration rate. Once albuminuria/proteinuria appears, renal function progressively declines and end-stage renal disease develops in a considerable number of patients.

## 8. Associated Factors and Possible Pathogenic Mechanisms

Before discussing the theoretical pathogenic mechanisms that could take part in DNND we will summarize the pathogenesis of DN, not completely understood. The most accepted theory is that metabolic pathways activated by hyperglycemia, hemodynamic factors, and oxidative stress are key players in the genesis of diabetic kidney disease. A variety of growth factors and cytokines are then induced through complex signal transduction pathways. Current research has focused on the podocyte as a central target for the effects of the metabolic milieu in the development and progression of diabetic albuminuria. Podocyte-derived vascular endothelial growth factor (VEGF), a permeability and angiogenic factor whose expression is increased in diabetic kidney disease, is perhaps a major mediator of the increased protein filtration. Angiotensin II stimulates podocyte-derived VEGF, suppresses nephrin expression, and induces TGF-β1 leading to podocyte apoptosis and fostering the development of glomerulosclerosis [35].

An alternative explanation for the development of proteinuria in DN, not necessary exclusive of the altered properties of glomerular ultrafiltration barrier, involves primarly an abnormality in tubular handling of ultrafiltered proteins.

### 8.1. Abnormal Glucose Metabolism

When Herzenberg in 1999 [3] reviewed 27 reported cases of ING he found that 55% met criteria for impaired glucose metabolism, frank diabetes or diabetic retinopathy. They discarded these cases and applied the term “ING” only to the cases with nodular sclerosis and no evidence of IGM. Due to this definition, since then, the majority of authors have dismissed ING cases with some kind of IGM not proper DM. In the same way, cases that have been diagnosed as diabetic retinopathy but did not meet DM analytic criteria are referred in the majority as DN [36,37] even though they would not develop DM until as late as nine years after. This fact was left aside from this analysis cases described as diabetic nephropathy very similar to those referred as ING with impaired fasting glucose at diagnosis according to the current criteria.

This might be the cause of the high heterogeneity observed in the size of the IGM effect in this revision. Following the current criteria, the observed frequency is of 57.8%, the IGM has a pooled prevalence of 74% (95% CI: 31% to 100%, I^2^ = 91%), but a maximum heterogeneity in the results of the different series, with two of them being 100% of the patients.

We have only found two cases with diabetic retinopathy at the diagnosis, one of them without IGM and a non-specified follow-up duration [22], and the other with normal glycemia, impaired OGTT [4] at the diagnosis, without further monitoring [36].

We think that having RT is not likely to make a difference between different cases of nephropathy, because both microvascular complications are thought to have common etiopathogenic mechanisms and diabetic retinopathy cases without DN have also been described.

Moreover, despite the fact that diabetes diagnostic criteria are biochemical and are well established, for the categories of increased risk for diabetes (prediabetes) too, Insulin Resistance (IR) needs a more sophisticated test for its diagnosis, which is readily available or for which many nephrologists are not familiar.

One of the most frequent causes of IR, apart from diabetes, is obesity, as well as the metabolic syndrome, very prevalent in published ING cases.

This theory is perfectly addressed by Filippone [27] who recently presented a case illustrating the possible role of IR in DNND development in the absence of glucose intolerance. This author describes the case of a normotensive, non-smoker, obese woman with normal glycemia and TTOG in which the Homeostasis model assessment of IR (HOMA-IR) was high. This led to the diagnosis of a metabolic syndrome. These authors discuss in a very interesting way about the possible mechanisms by which IR could be responsible for DN appearance in the absence of hyperglycemia. Evidence supports a role for the IR degree as one determining factor for developing DN in type 1 and type 2 DM. The important role of podocytes, recently pointed out in the pathogenesis of DN, which needs proper insulin signaling for correct functioning, reinforces this theory. A lower podocyte density per glomerulus has been shown in proteinuric DM patients in comparison to normoalbuminuric diabetic controls.

We agree with this analysis that many similar patients with DNND may have underlying IR. For all these reasons we think that probably the main base from which DNND can develop would be an IGM that includes any of its types, from resistance to insulin to diabetes.

We strongly encourage testing IR state in every patient diagnosed with DNND and performing kidney biopsy to patients with proteinuria and who have been confirmed with IR.

That could help early diagnosis of this entity and understanding its development and treatment. Should this theory be proved, it would be advisable to make CKD screening to patients with IR.

Probably other factors interact and potentiate those of IR in the appearance of DNND, especially the presence of hypertension, smoking and hypercholesterolemia.

### 8.2. Hypertension

In 1999 when Herzenberg [3] coined the term of ING he found hypertension in half of the patients who met diagnostic criteria. The causative role of hypertension in the development of DNND has been pointed out since then. The frequency of hypertension at the time of renal biopsy in all issued cases thereafter is much higher at 89.5%, practically in all cases of long duration. As follows from the results of the multivariate analysis of the published series, hypertension is the most important factor or cofactor to suffer ING, with a pooled prevalence of 91% (95% CI: 83% to 97%, I^2^ = 0%) homogeneous across all series. Moreover, it is the only one of the clinical factors associated with worse renal prognosis with an OR of 3.75 (95% CI: 0.9–15) *p* = 0.063. Markowitz [8] found that the arterial hypertension was the only independent factor associated with End-Stage Renal Disease in a multivariate study in his series of 23 cases with ING. These authors point out that the DNND could be a unique form of hypertensive arterionephrosclerosis that is modified by smoking.

In summary, it seems obvious that high blood pressure plays a major pathogenic role in the development of DNND. First, hypertension is highly prevalent. Second, hypertension is typically long-standing. Third, hypertension is a well established promoter of arteriosclerosis. Finally, hypertension is a previously established independent risk factor for the development of ESRD.

Pathogenetic mechanisms, through which hypertension could trigger DNND, could converge with those of DN and metabolic syndrome. Basic, clinical and population research indicate that visceral obesity, the main driver for type 2 diabetes and metabolic syndrome, raises blood pressure [38]. Visceral, but not subcutaneous, obesity appears to induce hypertension initially by increasing renal tubular reabsorption and causing a hypertensive shift of renal-pressure natriuresis through multiple mechanisms including activation of the sympathetic nervous system and RAAS, as well as physical compression of the kidneys [39]. The hypertension that ensues, as well as the increases in intraglomerular capillary pressure, and GFR, and the metabolic abnormalities (e.g., dyslipidemia, hyperglycemia) likely interact to cause renal injury. A similar sequence of events may lead to renal injury in diabetes, irrespective of obesity, suggesting that hypertension plays a key role in obesity and diabetes-associated renal injury. Further supporting the central role of hypertension in renal injury is the fact that progressive renal injury only occurs when hypertension is superimposed on obesity or diabetes.

Segmental glomerulosclerosis is a prominent feature in the so-called hypertensive nephrosclerosis where it appears to be the lesion causing renal failure [40]. It is thought that some other factor in the IR syndrome that so commonly accompanies hypertension is responsible for this type of renal lesion [41]. Some important studies in non-diabetic subjects support the hypothesis that IR plays a major role in renal failure attributed to hypertension [39,42]. Whether the IR can mediate different responses in kidney as DNND or segmental glomerulosclerosis, through the same or different mechanisms needs to be answered.

### 8.3. Tobacco Use

In the first published series on 23 patients with ING [8], authors found a frequency of smoking of 91%, significantly greater than in the general population (24%), with a prolonged duration of smoking. These findings were soon confirmed by others [9,10,20,23], however the frequency of smoking of total reported cases is 64% and our meta-analysis found a pooled prevalence of tobacco use of 76% (95% CI: 57% to 92%, I^2^ = 62%) with a moderate heterogeneity in different series, not associated to the race.

Even more striking was that the same group [13] found a much worse renal prognosis in current smokers compared with the patients who were nonsmokers or former smokers. In the univariate study that we performed, tobacco was not associated with worse prognosis, OR of 0.8 (95% CI: 0.3–2.4) *p* = 0.66, although we only knew if the patients were currently smoking or not.

Possible pathophysiological mechanisms by which smoking could promote DNND have been extensively discussed by Nashr *et al.* [13] and included the formation of advanced glycation products (AGE), induction of oxidative stress, angiogenesis and altered intrarenal hemodynamics.

Liang *et al.* [20], besides ING pattern found in four out of 10 long-term smokers describes other possible renal glomerular lesions associated with tobacco use like glomerulomegaly, focal and segmental glomerulosclerosis and glomerular ischemic changes.

An interesting recent study [26] has found a distinct pattern of injury observed in a small subset of heavy smokers characterized by findings of ING and a benign form of anti-GBM glomerulonephritis, with strong linear anti-GBM staining on immunofluorescence studies and occasional crescent formation in some glomeruli. Circulant anti-GBM antibodies could only be detected in one out of the three patients described. However, in Markowitz series [8] anti-GBM antibodies were detectable in 9% of patients with ING but without any description of crescents. Some authors [26] highlight the important association between smoking, endothelial injury, glomerular expansion and crescent formation and they encourage the need of a careful search for cellular crescents when strong intense linear IgG is observed along the GBM in ING cases. The fact that this pattern of dual injury is not observed in all heavy smoker patients suggests that other factors are necessary to develop such complication.

### 8.4. Overweight-Obesity

While the definition of ING a higher frequency of overweight/obesity was already patent in the few patients in which this information was available, 11 out of 16, the small number of reports with accurate data did not allow us to gauge the prevalence and severity of this phenomena.

The first important series which pointed out the possible role of obesity as a risk factor for the development of DNND was Li, *et al.* [9] who found in 15 ING cases 60% obesity and 27% overweight.

In all the cases published to date, the overweight presence is estimated at 32% and the obesity presence at 37%, without noting any effect on the renal prognosis. The pooled prevalence of overweight-obesity is 81.5% (95% CI: 51% to 100%, I^2^ = 0%) with a high heterogeneity between different studies.

The high proportion of overweight among patients with DNND would support the possible role of IR in the genesis of this illness.

The pathophisiology of obesity related and type 2 diabetes-related renal disease is almost identical. Common initiating events include interactions among multiple metabolic and hemodynamic factors that activate intracellular signaling pathways that in turn trigger the production of cytokines and growth factors, leading to renal disease. One of the earliest renal changes in obese humans is increased glomerular filtration rate (GFR). A likely explanation for increased GFR in obesity is increased salt reabsorption by the proximal tubule or loop of Henle, leading to tubuloglomerular feedback mediated reduction in afferent arteriolar resistance, increased intraglomerular capillary pressure and increased GFR. The increased GFR initially serves as a compensatory response that permits restoration of salt balance despite continued increases in tubular reabsorption but, over the long-term, contributes to renal injury, especially when combined with elevated blood pressure [43].

There are many similarities in the histological appearance of glomeruli from diabetic and obese individuals and both have early structural changes in the kidney that accompany hyperfiltration and albuminuria. Obesity-associated renal injury is characterized by glomerulomegaly, mesangial expansion, and podocytopenia leading to focal glomerulosclerosis [44]. Given these similarities, it is not surprising that the mechanisms leading to these changes are also similar. Diabetes and obesity are both states of low-grade inflammation associated with macrophage infiltration into the adipose tissue and the kidney. The infiltrating macrophages become a source of pro-inflammatory cytokines. Furthermore, increased adiposity triggers the release of adipokines into the circulation that in turn may cause renal injury via production of reactive oxygen species [45].

Kidneys of obese individuals often have glomerular/mesangial lipid deposits (foam cells) present, which supports the concept of lipotoxicity, *i.e.*, lipid-induced renal injury [44]. One of the mechanisms by which hyperlipidemia promotes glomerular injury is through renal upregulation of sterol-regulatory element-binding proteins, which in turn promotes podocyte apoptosis and mesangial cell proliferation and cytokine synthesis. Kimmelstein and Wilson [1] in their description of nodular glomerulosclerosis also showed lipid deposits in the diabetic kidney.

### 8.5. Metabolic Syndrome

In 2007 a Japanese series of seven patients with ING was published [19]. All the patients presented metabolic syndrome suggesting that individuals with metabolic syndrome are at high risk of developing CKD through unclear pathogenic mechanisms. In the present analysis we did not find sufficient data to make a true estimate of the prevalence of metabolic syndrome according to the most accepted criteria. Waist circumference, and lipid profiles of patients were not generally available. So we decided to analyze the coexistence of three criteria of metabolic syndrome according to WHO: IGM with hypertension and obesity, in this case using ethnic-specific end-points for estimating cardiovascular risk. We are therefore confident that our review has significantly underestimated the true prevalence of metabolic syndrome in DNND patients. That is why the pooled prevalence of metabolic syndrome is 64.4% (95% CI: 20% to 100%, I^2^ = 86%) with an extremely high heterogeneity between different studies. Yet 37% subjects meet all three criteria and patients with metabolic syndrome thus considered had worse prognosis than those who do not comply.

Individuals with metabolic syndrome are at high risk of developing CKD. An independent association between metabolic syndrome and albuminuria or proteinuria and CKD have been reported [45,46]. Since the metabolic syndrome is by definition a clustering of several metabolic factors and hypertension, it is often difficult to separate the effects of each element on glomerular hemodynamics and progression of renal injury. In a recent meta-analysis the strength of association seems to be increased as the number of components of metabolic syndrome increased. The most potent individual component that is associated with the CKD is hypertension OR 1.61 (95% CI: 1.29–2) while impaired fasting glucose 1.19 (95% CI 1.04–1.34).

Experimental studies [47], however, suggest that elevations in blood pressure exacerbate obesity-related glomerular hyperfiltration and albuminuria, further supporting the concept of an additive, or perhaps synergistic, effect of various components of obesity, metabolic syndrome, diabetes and hypertension on glomerular hemodynamics.

As we have described above, the possible role of IR in the triggering of DNND has been recently speculated about. Today, the central role of IR in the pathogenesis of metabolic syndrome cannot be denied. Several lines of evidence suggest a pathogenic role of IR on kidney dysfunction. Potential mechanisms are mostly due to the effect of single abnormalities related to insulin resistance and clustering into the metabolic syndrome. Hyperinsulinemia, which is inevitably associated to insulin resistance in non-diabetic states, also appears to play a role in kidney function by inducing glomerular hyperfiltration and increased vascular permeability. More recently, adipocytokines which are linked to insulin resistance, low grade inflammation, endothelial dysfunction and vascular damage have been proposed as additional molecules able to modulate kidney function. In addition, recent evidence points also to a role of insulin resistance at the level of the podocyte, an important player in early phases of diabetic kidney damage.

A great number of epidemiological studies have repeatedly reported the association between IR and kidney dysfunction in both non-diabetic [42,45] and diabetic subjects [48,49]. Among these, studies addressing the impact of IR genes on kidney dysfunction have played an important role to help establish a cause-effect relationship between these two traits [50].

Finally, intervention studies have shown that a favourable modulation of insulin resistance has a positive effect also on urinary albumin and total protein excretion [51].

Some animal models suggest an accelerated renal lesion through glucolipotoxicity on the podocytes and on the glomerular filtration membrane.

In conclusion, several data of different nature consistently support the role of insulin resistance and related abnormalities on kidney dysfunction. Intervention trials designed to investigate whether treating insulin resistance ameliorates also hard renal end-points are both timely and needed.

## 9. Conclusions and Future Trends

We believe that this review has several important limitations. The results of this review should be interpreted with caution due to the fact that the case series included are a poor source of original data. This pathology is so rare that there are no large series or other type of studies (cohort or case-control studies) available in the literature.

The DNND is a histological diagnosis classically defined as ING. We believe that DNND is a spectrum of disease in which nodular glomerulosclerosis is just a stage, so a limitation of our study is not taking into account other stages of this disease.

As ING is a histological finding rarely suspected, the data search is retrospective in most of the published cases, causing a loss of important data, for example HbA1c. The classification of the patients according to IGM was made with a heterogeneous database compiled with available data.

The definition of the metabolic syndrome in our paper is biased because we used IGM considered as we explained earlier, the BMI instead of waist circumference in most cases and in some cases we just considered the diagnosis referred by the authors even if it was not sufficiently documented.

From our results and others’ it seems that DNND is favoured by recognized cardiovascular risk factors. Aparently one single risk factor is not enough to cause DNND. Data from basic and clinical studies suggest that obesity, hypertension, hyperglycemia, hyperlipidemia and other elements of the metabolic syndrome are highly interrelated and contribute to the development and progression of diabetic nephropathy [52]. Given their close relationship, it is often difficult to dissect out their individual effects. It is likely that multiple metabolic abnormalities act in concert to initially cause renal vasodilation, glomerular hyperfiltration and albuminuria that, in turn, effect glomerular pathology, especially when combined with increased blood pressure, and that ultimately progresses to diabetic nephropathy. Other factors not commented in the paper as genetic and environmental factors could play a role or interact. The most plausible hypothesis for the ocurrence of DNND would be a special form of atherosclerotic or metabolic glomerulopathy than can occur in the context of diabetes or not. Clinical spectrum of cardiovascular risk factors and histological findings support this theory. One of their characteristic clinical features is hypertension.

Regarding all this, a central role for IR in the pathogenesis of DNND appears to be the most plausible explanation and link between both entities, DN and DNND. Identification of the causes and pathogenic mechanisms of DNND requires further studies.
